# Supplementary material for: DNA shuffling to improve crude-water interfacial activity in biosurfactants with OmpA protein of Escherichia coli
Source: PeerJ. 2024 Dec 3;12:e17239. doi: 10.7717/peerj.17239 (PMC11623092; doi:10.7717/peerj.17239)
Supplement: Supplemental Information 1 [file peerj-12-17239-s001.pdf]

Supporting material

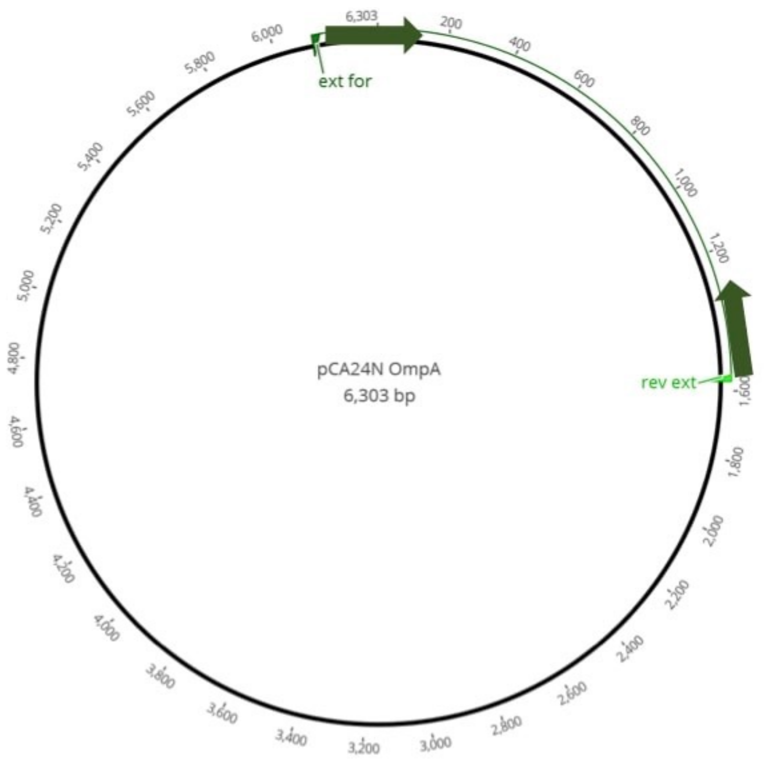

Figure 1s. Amplification region for OmpA in pCA24N plasmid

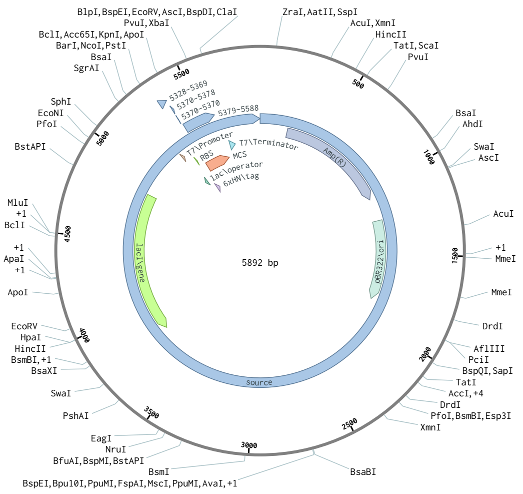

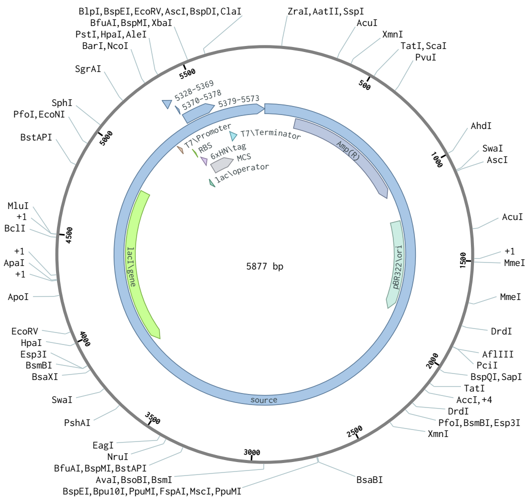

Figure 2s. pET6xHN-N with mutant 6 and 12

Purification of plasmid pCA24N with *ompA* gene.

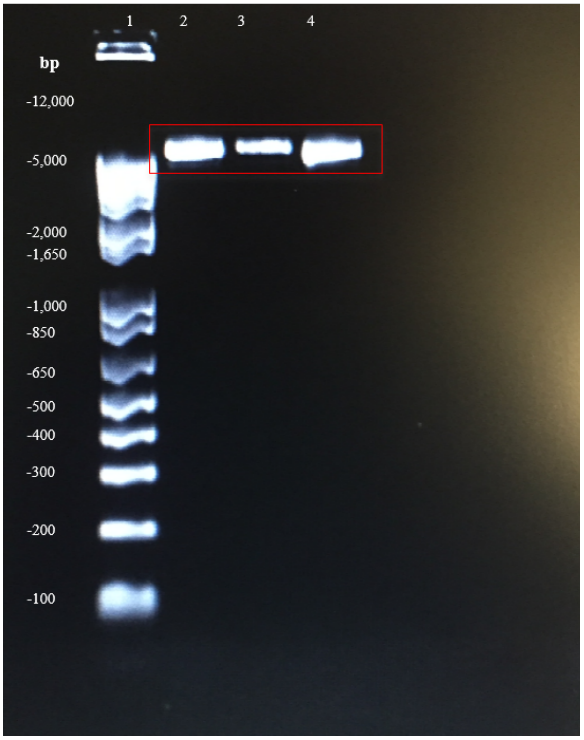

Figure 3s Plasmid pCA24N purified

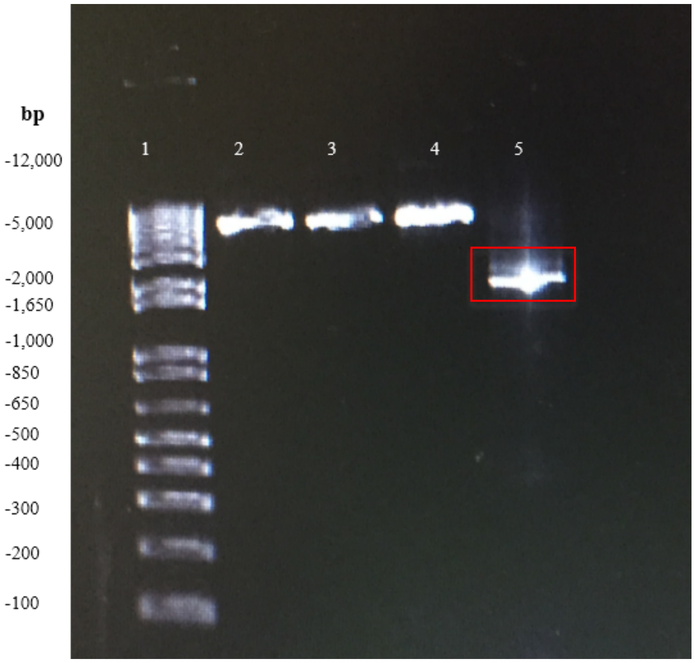

Figure 4s. Lane 1) 1 Kb DNA ladder, Lane 2) 3) and 4) plasmid pCA24N with *ompA* Lane 5) amplification with primers EF and 3ER

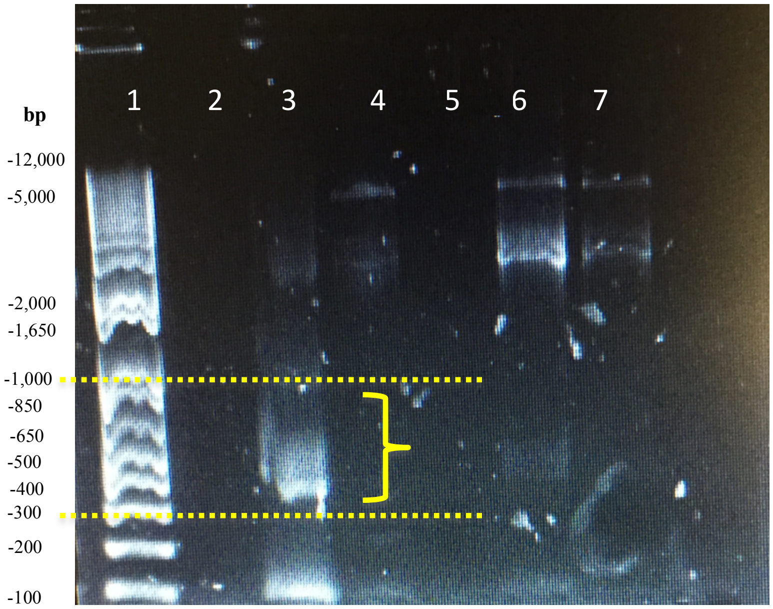

Figure 5s. Random fragmentation with DNase I. Lane 1) 1 Kb DNA ladder, 2) Reaction for 2 minutes, 3) Reaction for 30 seconds, 4) Reaction for 60 seconds, 5) Reaction for 90 seconds, 6) Reaction for 5 seconds, 7) reaction for 10 seconds.

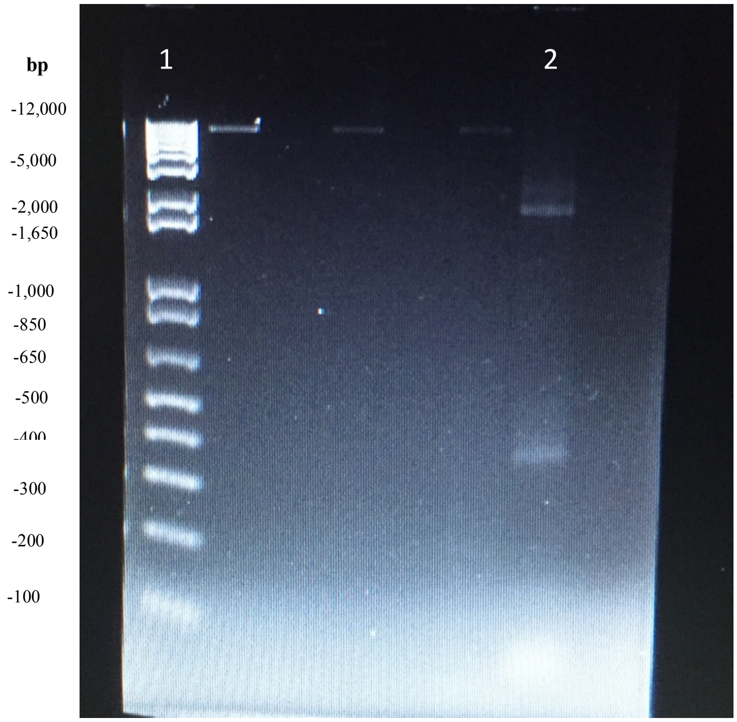

Figure 6s. Reassembly with inner primers 3IR and 3IF. Lane 1) 1 Kb DNA ladder, Lane 2) Fragments after reassembly.

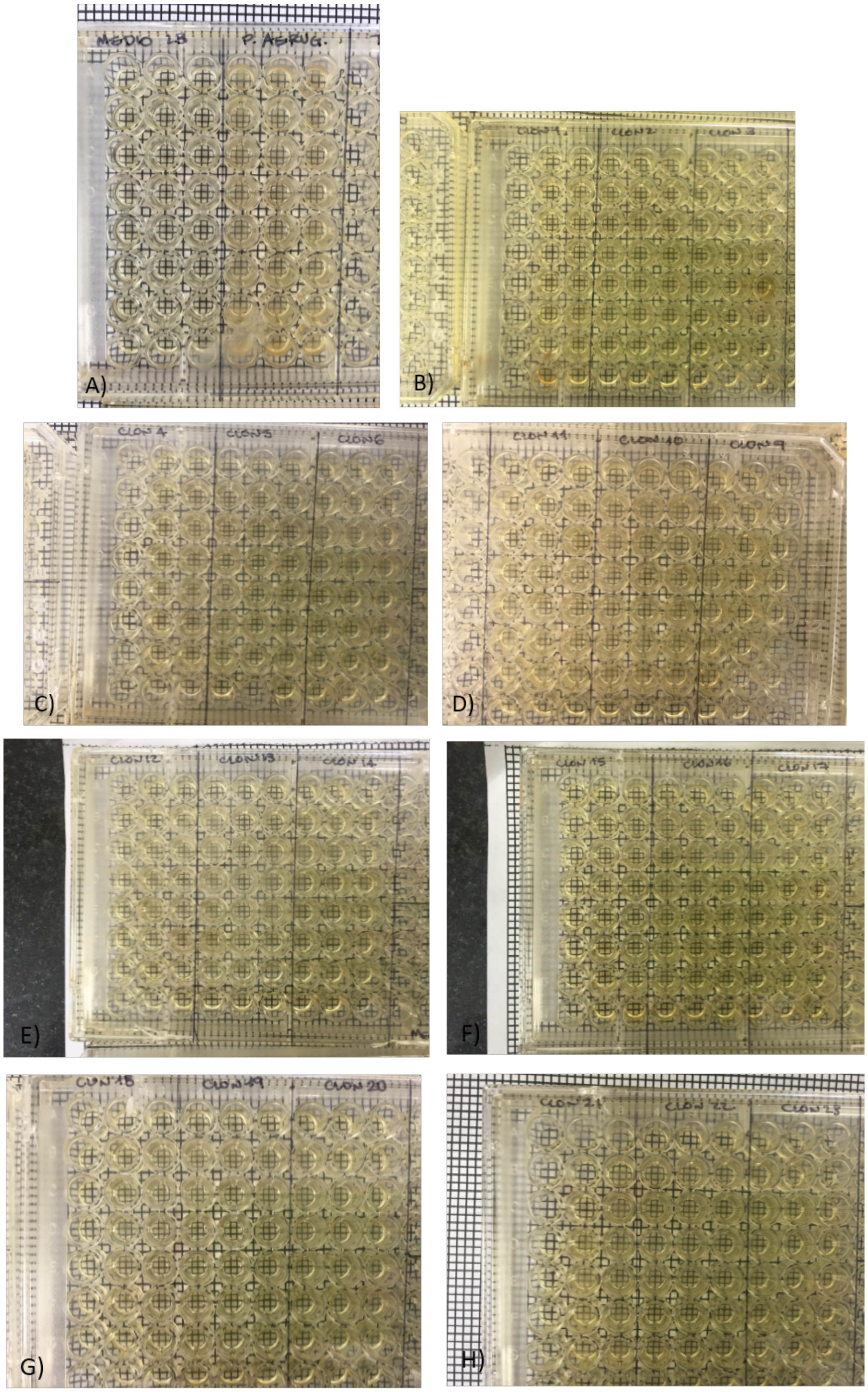

Figure 7s. 96-well plates to evaluate ability to modify IFT of each clone. A) Positive controls, LB medium and supernatant of Pseudomonas aeruginosa, B) Supernatant for clones 1, 2 and 3, C) Supernatant for clones 4, 5 and 6, D) Supernatant for clones 9, 10 and 11, E) Supernatant for clones 12, 13 and 14, F) Supernatant for clones 15, 16 and 17, G) Supernatant for clones 18, 19 and 20, H) Supernatant for clones 21, 22 and 23.

Pairwise Sequence Alignments

Clone 1

```
#=======================================

OmpA325minimd      1 GCGCCGAAAGATAACACCTGGTATACCGGCGCGAAACTGGGCTGGAGCCA     50

Clon               1 --------------------------------------------------      0

OmpA325minimd     51 GTATCATGATACCGGCTTTATTAACAACAACGGCCCGACCCATGAAAACC    100

Clon               1 --------------------------------------------------      0

OmpA325minimd    101 AGCTGGGCGCGGGCGCGTTTGGCGGCTATCAGGTGAACCCGTATGTGGGC    150

Clon               1 --------------------------------------------------      0

OmpA325minimd    151 TTTGAAATGGGCTATGATTGGCTGGGCCGCATGCCGTATAAAGGCAGCGT    200

Clon               1 --------------------------------------------------      0

OmpA325minimd    201 GGAAAACGGCGCGTATAAAGCGCAGGGCGTGCAGCTGACCGCGAAACTGG    250

Clon               1 --------------------------------------------------      0

OmpA325minimd    251 GCTATCCGATTACCGATGATCTGGATATTTATACCCGCCTGGGCGGCATG    300
```

```
Clon               1 --------------------------------------------------      0

OmpA325minimd    301 GTGTGGCGCGCGGATACCAAAAGCAACGTGTATGGCAAAAACCATGATAC    350

Clon               1 --------------------------------------------------      0

OmpA325minimd    351 CGGCGTGAGCCCGGTGTTTGCGGGCGGCGTGGAATATGCGATTACCCCGG    400

Clon               1 --------------------------------------------------      0

OmpA325minimd    401 AAATTGCGACCCGCCTGGAATATCAGTGGAC--CAACA----ACATTGGC    444
                     |||   ||  |||||        |.|||.||  |||||    .||||
Clon               1 AAA---CG--CCGCC--------CGGTGAACTGCAACACCACCCATT---     34

OmpA325minimd    445 GAT-GCGCATACCATTGGCACCCGCCCGGATAACGGCATGCTGAGCC--T    491
                      || |||| .|.||||      ||||||      .||||.|   |||  |
Clon              35 -ATCGCGC-GAACATT------CGCCCG------CGCATTC---GCCCGT     67

OmpA325minimd    492 GGGCGTGAGCTATCGCTTTGGCCAGGGCGAAGCGGCGCCGGTGGTG----    537
                     ||||||| || ||.||.||   |.|.|||||         .|.|||
Clon              68 GGGCGTG-GC-ATAGCATT---CTGTGCGAA---------ATTGTGATTC    103

OmpA325minimd    538 GC-GCCGGCGCCGGCGCCGGCGCCGGAA-GTGCA-GACCAAACATTTTAC    584
                     || |||.|.||   |||...||||.||| .|||| ||          ||.
Clon             104 GCAGCCAGGGC---CGCATTCGCCTGAACCTGCAGGA----------TAG    140

OmpA325minimd    585 CCTGAAAAGCGATGTGCTGTTTAACTTTAACAAAGCGACCCTGAAACCGG    634
                     ||||         |..|||.||                  ||||
Clon             141 CCTG---------GGCCTGATT------------------CTGA------    157

OmpA325minimd    635 AAGGCCAGGCGGCGCTGGATCAGCTGTATAGCCAGCTGAGCAACCTGGAT    684
                        |||.||||          |||||           |||   ||||..|
Clon             158 ---GCCTGGCG----------AGCTG-----------GAG---CCTGTTT    180

OmpA325minimd    685 CCGAAAGATGGCAGCGTGGTGGTGCTGGGCTATACCGATCGCATTGGCAG    734
                     |||
Clon             181 CCG-----------------------------------------------    183

OmpA325minimd    735 CGATGCGTATAACCAGGGCCTGAGCGAACGCCGCGCGCAGAGCGTGGTGG    784

Clon             184 --------------------------------------------------    183

OmpA325minimd    785 ATTATCTGATTAGCAAAGGCATTCCGGCGGATAAAATTAGCGCGCGCGGC    834

Clon             184 --------------------------------------------------    183

OmpA325minimd    835 ATGGGCGAAAGCAACCCGGTGACCGGCAACACCTGCGATAACGTGAAACA    884

Clon             184 --------------------------------------------------    183

OmpA325minimd    885 GCGCGCGGCGCTGATTGATTGCCTGGCGCCGGATCGCCGCGTGGAAATTG    934

Clon             184 --------------------------------------------------    183

OmpA325minimd    935 AAGTGAAAGGCATTAAAGATGTGGTGACCCAGCCGCAGGCG    975

Clon             184 -----------------------------------------    183
```

Clone 5

```
#=======================================

OmpA325minimd      1 GCGCCGAAAGATAACACCTGGTATACCGGCGCGAAACTGGGCTGGAGCCA     50

Clon               1 --------------------------------------------------      0

OmpA325minimd     51 GTATCATGATACCGGCTTTATTAACAACAACGGCCCGACCCATGAAAACC    100

Clon               1 --------------------------------------------------      0

OmpA325minimd    101 AGCTGGGCGCGGGCGCGTTTGGCGGCTATCAGGTGAACCCGTATGTGGGC    150

Clon               1 --------------------------------------------------      0

OmpA325minimd    151 TTTGAAATGGGCTATGATTGGCTGGGCCGCATGCCGTATAAAGGCAGCGT    200

Clon               1 --------------------------------------------------      0

OmpA325minimd    201 GGAAAACGGCGCGTATAAAGCGCAGGGCGTGCAGCTGACCGCGAAACTGG    250
```

```
Clon               1 --------------------------------------------------      0

OmpA325minimd    251 GCTATCCGATTACCGATGATCTGGATATTTATACCCGCCTGGGCGGCATG    300

Clon               1 --------------------------------------------------      0

OmpA325minimd    301 GTGTGGCGCGCGGATACCAAAAGCAACGTGTATGGCAAAAACCATGATAC    350

Clon               1 --------------------------------------------------      0

OmpA325minimd    351 CGGCGTGAGCCCGGTGTTTGCGGGCGGCGTGGAATATGCGATTACCCCGG    400
                                                            |||..||
Clon               1 ---------------------------------------GATGGCC----      7

OmpA325minimd    401 AAATTGCGACCCGCCTGGAATATCAGTGGACCAACAACATTGGCGATGCG    450
                     |.||||.||..||||||                  |.|||||||    ||
Clon               8 AGATTGTGATTCGCCTG------------------ACCATTGGC----CG     35

OmpA325minimd    451 CATACCATTGGCACCCGCCCGGATAACGGCATGCTGAGCCTGGGCGTGAG    500
                     |||...|||              ||.||||
Clon              36 CATTGAATT--------------TAGCGGC--------------------     51

OmpA325minimd    501 CTATCGCTTTGGCCAGGGCGAAGCGGCGCCGGTGGTGGCGCCGGCGCCGG    550
                                     .|||||...||||.||
Clon              52 ----------------CGCGAATTTGCGCTGG------------------     67

OmpA325minimd    551 CGCCGGCGCCGGAAGTGCAGACCAAAC-ATTTACCCTGAAAAGCGATGT    599
                              ||||          .||| |.|||||||.|||.|||...||
Clon              68 ---------CGGA----------TAACAACTTTACCCAGAACAGCCGCGT     98

OmpA325minimd    600 GCTGTTTAACTTTAACAAAGCGACCCTGAAACCGGAAGGCCAGGCG-GCG    648
                     ||.||                                 |||   || |||
Clon              99 GCCGT---------------------------------GCC---CGCGCG    112

OmpA325minimd    649 CTGGATCAGCTGTATAGCCAGCTGAGCAACCTGGATCCGAAAGATGGC-A    697
                     |  ||.|||||.||             ||||              ||| .
Clon             113 C--GAACAGCTTTA-------------AACC--------------GGCGG    133

OmpA325minimd    698 GCGTGGTGGTGCTGGGCTATACCGATCGCATTGGCAGCGATGCGTAT-AA    746
                     ||.|||||..|||||                         ||||..| ||
Clon             134 GCCTGGTGCCGCTGG-------------------------TGCGCGTGAA    158

OmpA325minimd    747 CCAGGGCCTGAGCGAACGCCGCGCGCAGAGCGTGGTGGATTAT-CTGATT    795
                     |         ||||||         |.|.||||    |||||| .|||||
Clon             159 C---------AGCGAA---------CTGGGCGT----GATTATGGTGATT    186

OmpA325minimd    796 AGCAAAGGCATTCCGGCGGATAAAATTAGCGCGCGCGGCATGGGCGAAAG    845
                                    ||||      |.|||
Clon             187 ---------------GCGG------TGAGC--------------------    195

OmpA325minimd    846 CAACCCGGTGACCGGCAACACCTGCGATAACGTGAAACAGCGCGCGGCGC    895

Clon             196 --------------------------------------------------    195

OmpA325minimd    896 TGATTGATTGCCTGGCGCCGGATCGCCGCGTGGAAATTGAAGTGAAAGGC    945

Clon             196 --------------------------------------------------    195

OmpA325minimd    946 ATTAAAGATGTGGTGACCCAGCCGCAGGCG    975

Clon             196 ------------------------------    195
```

Clone 6

```
#=======================================

OmpA325minimd      1 GCGCCGAAAGATAACACCTGGTATACCGGCGCGAAACTGGGCTGGAGCCA     50

Clon               1 --------------------------------------------------      0

OmpA325minimd     51 GTATCATGATACCGGCTTTATTAACAACAACGGCCCGACCCATGAAAACC    100
```

```
Clon                1 --------------------------------------------------      0

OmpA325minimd     101 AGCTGGGCGCGGGCGCGTTTGGCGGCTATCAGGTGAACCCGTATGTGGGC    150

Clon                1 --------------------------------------------------      0

OmpA325minimd     151 TTTGAAATGGGCTATGATTGGCTGGGCCGCATGCCGTATAAAGGCAGCGT    200

Clon                1 --------------------------------------------------      0

OmpA325minimd     201 GGAAAACGGCGCGTATAAAGCGCAGGGCGTGCAGCTGACCGCGAAACTGG    250

Clon                1 --------------------------------------------------      0

OmpA325minimd     251 GCTATCCGATTACCGATGATCTGGATATTTATACCCGCCTGGGCGGCATG    300

Clon                1 --------------------------------------------------      0

OmpA325minimd     301 GTGTGGCGCGCGGATACCAAAAGCAACGTGTATGGCAAAAACCATGATAC    350
                                                                ||.||.||
Clon                1 ------------------------------------------CAGGAAAC      8

OmpA325minimd     351 CGGCG-TGAGCCCGGTGTTTGCGGGCGGCGTGGAATATGCGATTACCCCG    399
                      | ||| |||.|                       ||    ||||||||||
Clon                9 C-GCGATGACC-----------------------AT----GATTACCCCG     30

OmpA325minimd     400 GAAATTGCGACCCGCCTGGAATATCAGTGGACCAACAACATTGGCGATGC    449
                                            |.|||.|.||         ||.|..||
Clon               31 ----------------------AGCAGCGAAC---------TGACCCTG-     48

OmpA325minimd     450 GCATACCATTGGCACCCGCCCGGATAACGGCATGCTGAGCCTGGGCGTGA    499
                          ||||..||||||.||||||    |||        |||||
Clon               49 ----ACCAAAGGCACCAGCCCGG----CGG--------GCCTG-------     75

OmpA325minimd     500 GCTATCG-CTTTGGCCAGGGCGAAGCGGCGCCGGTGGTG-GCGCCGGCGC    547
                         |.|| .||||                  ||.||||| |||     ||
Clon               76 ---AACGAATTTG------------------CGCTGGTGAGCG-----GC     99

OmpA325minimd     548 CGGCGCCGGCGCCGGAAGTGCAGACCAAACATTT--TACCCTGAAAAGCG    595
                      |.|                            |||  ||||      |||.
Clon              100 CAG----------------------------TTTCATACC------AGCC    115

OmpA325minimd     596 ATGTGCTGTTTAACTTTAACAAAGCGACCCTGAAACCGGAAGGCCAGGCG    645
                      ..||||.||                                 |||   ||
Clon              116 GCGTGCCGT---------------------------------GCC---CG    129

OmpA325minimd     646 -GCGCTGGATCAGCTGTATAGCCAGCTGAGCAACCTGGAT----CCGAAA    690
                       ||||  ||.||||     .|||.|||||.||    |.||    ||||..
Clon              130 CGCGC--GAACAGC-----CGCCCGCTGAACA----GCATTCGCCCGATT    168

OmpA325minimd     691 GATG-GCAGCGTGGTGGTGCTGGGCTATACCGATCGCATTGGCAGCGATG    739
                      | || ||.||.|               ||||..|  |||||||  ||| |
Clon              169 G-TGAGCCGCAT---------------TACCATT--CATTGGC--CGA-G    197

OmpA325minimd     740 CGTATAACCAGGGCCTGAGCGAACGCCGCGCGCAGAGCGTGGTGGATTAT    789
                      |.|.||.
Clon              198 CTTTTAT-------------------------------------------    204

OmpA325minimd     790 CTGATTAGCAAAGGCATTCCGGCGGATAAAATTAGCGCGCGCGGCATGGG    839

Clon              205 --------------------------------------------------    204

OmpA325minimd     840 CGAAAGCAACCCGGTGACCGGCAACACCTGCGATAACGTGAAACAGCGCG    889

Clon              205 --------------------------------------------------    204

OmpA325minimd     890 CGGCGCTGATTGATTGCCTGGCGCCGGATCGCCCGCGTGGAAATTGAAGTG    939

Clon              205 --------------------------------------------------    204

OmpA325minimd     940 AAAGGCATTAAAGATGTGGTGACCCAGCCGCAGGCG    975

Clon              205 ------------------------------------    204
```

Clone 7

```
#========================================

OmpA325minimd        1 --------------------------------------------------      0

Clon                 1 TTTCATCAGCAGCAGTGGCCGGATGCGCTGGGCCATGATTGGGCGCTGGT     50

OmpA325minimd        1 --------------------------------------------------      0

Clon                51 GCTGCTGGTGGTGACCAGCCGCGAATTTCTGTGCGAAATTGTGATGAGCT    100

OmpA325minimd        1 --------------------------------------------------      0

Clon               101 ATGATTGGCTGGGCCGCATGCCGTATTTTGGCACCGTGGAAAACCAGGTG    150

OmpA325minimd        1 --------------------------------------------------      0

Clon               151 CTGGGCCTGCGCTGCATTCAGAGCAGCGGCCGCAGCACCGATCGCACCGG    200

OmpA325minimd        1 --------------------------------------------------      0

Clon               201 CCTGCCGAACCATCGCCCGGGCCATCTGCAGATTCGCCCGCCGTGGCATC    250

OmpA325minimd        1 --------------------------------------------------      0

Clon               251 TGTGCGAAATTGTGAAAAGCAACGTGTATGGCAAAAACCATGATACCGGC    300

OmpA325minimd        1 --------------------------------------------------      0

Clon               301 GCGAACCGCTATCGCGTGAAACTGCTGCCGATTAACATTGGCGATGCGCA    350

OmpA325minimd        1 -------------------GCGCCGAAAGATAACACCTGGTATACCGGCG     31
                                          |||||||||||||||||||||||||||||||
Clon               351 TACCATTGGCACCCGCCCGGCGCCGAAAGATAACACCTGGTATACCGGCG    400

OmpA325minimd       32 CGAAACTGGGCTGGAGCCAGTATCATGATACCGGCTTTATTAACAACAAC     81
                       |||||||||||||||||||||||    |.|||||              |.
Clon               401 CGAAACTGGGCTGGAGCCAGTAT----AAACCGG--------------AA    432

OmpA325minimd       82 GGCCCGACCCATGAAAACCAGCTGGGCGCGGGCGCGTTTGGCGGCTATCA    131
                       ||||.|.|                |||||
Clon               433 GGCCAGGC----------------GGCGC---------------------    445

OmpA325minimd      132 GGTGAACCCGTATGTGGGCTTTGAAATGGGCTATGATTGGCTGGGCCGCA    181
                                     ||||||||||||||||||||||||||||||||||||
Clon               446 --------------TGGGCTTTGAAATGGGCTATGATTGGCTGGGCCGCA    481

OmpA325minimd      182 TGCCGTATAAAGGCAGCGTGGAAAACGGCGCGTATAAAGCGCAGGGCGTG    231
                       ||||||||||||   ||..|||.||||||    ||
Clon               482 TGCCGTATAAAG---GCCCGGATAACGGC----AT---------------    509

OmpA325minimd      232 CAGCTGACCGCGAAACTGGGCTATCCGATTACCGATGATCTGGATATTTA    281
                         |||||.|      ||||||             .|||.||  ||
Clon               510 --GCTGAGC------CTGGGC-------------GTGAGCT--AT-----    531

OmpA325minimd      282 TACCCGCCTGGGCGGCATGGTGTGGCGCGCGGATACCAAAAGCAACGTGT    331
                           |||.|.|||  ||.||.|..|||       ||||||||||||||||
Clon               532 ----CGCTTTGGC--CAGGGCGAAGCG-------ACCAAAAGCAACGTGT    568

OmpA325minimd      332 ATGGCAAAAACCATGATACCGGCGTGAGCCCGGTGTTTGCGGGCGGCGTG    381
                       ||||||||||||||||||||||||||||||||||||||||||||||||||
Clon               569 ATGGCAAAAACCATGATACCGGCGTGAGCCCGGTGTTTGCGGGCGGCGTG    618

OmpA325minimd      382 GAATATGCGATTACCCCGGAAATTGCGACCCGCCTGGAATATCAGTGGAC    431
                       ||||||||||||||||||||||||||||||||||||||||||||||||||
Clon               619 GAATATGCGATTACCCCGGAAATTGCGACCCGCCTGGAATATCAGTGGAC    668

OmpA325minimd      432 CAACAACATTGGCGATGCGCATACCATTGGCACCCGCCCGGATAACGGCA    481
                       ||||||||||||||||||||||||||||||||||||||||||||||||||
Clon               669 CAACAACATTGGCGATGCGCATACCATTGGCACCCGCCCGGATAACGGCA    718
```

```
OmpA325minimd    482 TGCTGAGCCTGGGCGTGAGCTATCGCTTTGGCCAGGGCGAAGCGGCGCCG    531
                     ||||||||||||||||||||||||||||||||||||||||||||||||||
Clon             719 TGCTGAGCCTGGGCGTGAGCTATCGCTTTGGCCAGGGCGAAGCGGCGCCG    768

OmpA325minimd    532 GTGGTGGCGCCGGCGCCGGCGCCGGCGCCGGAAGTGCAGACCAAACATTT    581
                     ||||||||||||||||||||||||||||||||||||||||||||||||||
Clon             769 GTGGTGGCGCCGGCGCCGGCGCCGGCGCCGGAAGTGCAGACCAAACATTT    818

OmpA325minimd    582 TACCCTGAAAAGCGATGTGCTGTTTAACTTTAACAAAGCGACCCTGAAAC    631
                     ||||||||||||||||||||||||||||||||||||||||||||||||||
Clon             819 TACCCTGAAAAGCGATGTGCTGTTTAACTTTAACAAAGCGACCCTGAAAC    868

OmpA325minimd    632 CGGAAGGCCAGGCGGCGCTGGATCAGCTGTATAGCCAGCTGAGCAACCTG    681
                     ||||||||||||||||||||
Clon             869 CGGAAGGCCAGGCGGCGCTG------------------------------    888

OmpA325minimd    682 GATCCGAAAGATGGCAGCGTGGTGGTGCTGGGCTATACCGATCGCATTGG    731

Clon             889 --------------------------------------------------    888

OmpA325minimd    732 CAGCGATGCGTATAACCAGGGCCTGAGCGAACGCCGCGCGCAGAGCGTGG    781

Clon             889 --------------------------------------------------    888

OmpA325minimd    782 TGGATTATCTGATTAGCAAAGGCATTCCGGCGGATAAAATTAGCGCGCGC    831

Clon             889 --------------------------------------------------    888

OmpA325minimd    832 GGCATGGGCGAAAGCAACCCGGTGACCGGCAACACCTGCGATAACGTGAA    881

Clon             889 --------------------------------------------------    888

OmpA325minimd    882 ACAGCGCGCGGCGCTGATTGATTGCCTGGCGCCGGATCGCCGCGTGGAAA    931

Clon             889 --------------------------------------------------    888

OmpA325minimd    932 TTGAAGTGAAAGGCATTAAAGATGTGGTGACCCAGCCGCAGGCG    975

Clon             889 --------------------------------------------    888
```

Clone 12

```
#=======================================

OmpA325minimd      1 GCGCCGAAAGATAACACCTGGTATACCGGCGCGAAACTGGGCTGGAGCCA     50

Clon               1 --------------------------------------------------      0

OmpA325minimd     51 GTATCATGATACCGGCTTTATTAACAACAACGGCCCGACCCATGAAAACC    100

Clon               1 --------------------------------------------------      0

OmpA325minimd    101 AGCTGGGCGCGGGCGCGTTTGGCGGCTATCAGGTGAACCCGTATGTGGGC    150

Clon               1 --------------------------------------------------      0

OmpA325minimd    151 TTTGAAATGGGCTATGATTGGCTGGGCCGCATGCCGTATAAAGGCAGCGT    200

Clon               1 --------------------------------------------------      0

OmpA325minimd    201 GGAAAACGGCGCGTATAAAGCGCAGGGCGTGCAGCTGACCGCGAAACTGG    250
                                                       |||   |.|||||...
Clon               1 ----------------------------------CTG---GTGAAACGCC     13
```

```
OmpA325minimd    251 GCTATCCGATTACCGATGATCTGGATATTTATAC-CCGCCTGGGCGGCAT    299
                     ||          |||.|||.|||.|       || ||.||       |||
Clon              14 GC----------CCGGTGAACTGCA-------ACACCACC-------CAT     39

OmpA325minimd    300 GGTGTGGCGCGCGGATACCAAAAGCAACGTGTATGGCAAAAACCATGATA    349
                       |.|.|||||              |||.|   |.||
Clon              40 --TATCGCGCG--------------AACAT---TCGC-------------     57

OmpA325minimd    350 CCG-GCGTGAGCCCGGTGTTTGCGGGCGGCGTGGAATATGCGATTACCCC    398
                     ||| ||.|..|||| |||         |||||||.||| || |||   |.
Clon              58 CCGCGCATTCGCCC-GTG---------GGCGTGGCATA-GC-ATT---CT     92

OmpA325minimd    399 G---GAAATTGCGACCCGCCTGGAATATCAGTGGACCAACAACATTGGCG    445
                     |   |||||||.||..||          |||    |||       .|||
Clon              93 GTGCGAAATTGTGATTCG----------CAG----CCA-------GGGC-    120

OmpA325minimd    446 ATGCGCATAC-CATTGGCACCCGCCCGGATAACGGCATGCTGAGCCTGGG    494
                        |||||.| |.|  |.|||.| |.||||            ||||||||
Clon             121 ---CGCATTCGCCT--GAACCTG-CAGGAT------------AGCCTGGG    152

OmpA325minimd    495 CGTGAGCTATCGCTT-TG-GCCAGGGCGAAGCGGCGCCGGTGGTGGCGCC    542
                     |.|||        || || ||| .|||||            |.|||.|||
Clon             153 CCTGA--------TTCTGAGCC-TGGCGA------------GCTGGAGCC    181

OmpA325minimd    543 GGC-GCCGGCGCCGGCGCCGGAAGTGCAGACCAAACATTTTACCCTGAAA    591
                     .|| ||||
Clon             182 TGCTGCCG------------------------------------------    189

OmpA325minimd    592 AGCGATGTGCTGTTTAACTTTAACAAAGCGACCCTGAAACCGGAAGGCCA    641

Clon             190 --------------------------------------------------    189

OmpA325minimd    642 GGCGGCGCTGGATCAGCTGTATAGCCAGCTGAGCAACCTGGATCCGAAAG    691

Clon             190 --------------------------------------------------    189

OmpA325minimd    692 ATGGCAGCGTGGTGGTGCTGGGCTATACCGATCGCATTGGCAGCGATGCG    741

Clon             190 --------------------------------------------------    189

OmpA325minimd    742 TATAACCAGGGCCTGAGCGAACGCCGCGCGCAGAGCGTGGTGGATTATCT    791

Clon             190 --------------------------------------------------    189

OmpA325minimd    792 GATTAGCAAAGGCATTCCGGCGGATAAAATTAGCGCGCGCGGCATGGGCG    841

Clon             190 --------------------------------------------------    189

OmpA325minimd    842 AAAGCAACCCGGTGACCGGCAACACCTGCGATAACGTGAAACAGCGCGCG    891

Clon             190 --------------------------------------------------    189

OmpA325minimd    892 GCGCTGATTGATTGCCTGGCGCCGGATCGCCGCGTGGAAATTGAAGTGAA    941

Clon             190 --------------------------------------------------    189

OmpA325minimd    942 AGGCATTAAAGATGTGGTGACCCAGCCGCAGGCG    975

Clon             190 ----------------------------------    189
```

Clone 13

```
#=======================================

OmpA325minimd      1 GCGCCGAAAGATAACACCTGGTATACCGGCGCGAAACTGGGCTGGAGCCA     50

Clon               1 --------------------------------------------------      0

OmpA325minimd     51 GTATCATGATACCGGCTTTATTAACAACAACGGCCCGACCCATGAAAACC    100

Clon               1 --------------------------------------------------      0
```

```
OmpA325minimd    101 AGCTGGGCGCGGGCGCGTTTGGCGGCTATCAGGTGAACCCGTATGTGGGC    150

Clon               1 --------------------------------------------------      0

OmpA325minimd    151 TTTGAAATGGGCTATGATTGGCTGGGCCGCATGCCGTATAAAGGCAGCGT    200

Clon               1 --------------------------------------------------      0

OmpA325minimd    201 GGAAAACGGCGCGTATAAAGCGCAGGGCGTGCAGCTGACCGCGAAACTGG    250

Clon               1 --------------------------------------------------      0

OmpA325minimd    251 GCTATCCGATTACCGATGATCTGGATATTTATACCCGCCTGGGCGGCATG    300

Clon               1 --------------------------------------------------      0

OmpA325minimd    301 GTGTGGCGCGCGGATACCAAAAGCAACGTGTATGGCAAAAACCA-----T    345
                                             ||||   ||||      |||     |
Clon               1 ------------------------AACG---ATGG------CCAGATTGT     17

OmpA325minimd    346 GATACCGGCGTGAGCCCGGTGTTTGCGGGCGGCGTGGAATATGCGATTAC    395
                     |||.|  ||.|||  ||..|       |||.||.|.||||      |||.
Clon              18 GATTC--GCCTGA--CCATT-------GGCCGCATTGAAT------TTAG     50

OmpA325minimd    396 C--CCG-GAAATTGCG------------ACCCGCCTGGAATATCAGTGGA    430
                     |  ||| |||.|||||            ||   |||||||..||||||
Clon              51 CGGCCGCGAATTTGCGCTGGGCCATGGCAC---CCTGGAAATTCAGTG--     95

OmpA325minimd    431 CCAACAACATTGGCGATGCGCATAC-------CATTGGC----ACCCGCC    469
                         |.|.||| |.|||.||||..|       ||||.||    |||.| |
Clon              96 ----CGAAATT-GTGATTCGCAGCCAGGGCCGCATTCGCCTGAACCTG-C    139

OmpA325minimd    470 CGGATAAC---GGC---ATGCTGAGCCTGG---GCGTGAGCTATCGCTTT    510
                     .|||||.|   |||   ||.||||||||||   ||..||||   |..|||
Clon             140 AGGATAGCCTGGGCCTGATTCTGAGCCTGGCGAGCTGGAGC---CTGTTT    186

OmpA325minimd    511 GGCCAGGGCGAAGCGGCGCCGGTGGTGGCGCCGGCGCCGGCGCCGGCGCC    560

Clon             187 --------------------------------------------------    186

OmpA325minimd    561 GGAAGTGCAGACCAAACATTTTACCCTGAAAAGCGATGTGCTGTTTAACT    610

Clon             187 --------------------------------------------------    186

OmpA325minimd    611 TTAACAAAGCGACCCTGAAACCGGAAGGCCAGGCGGCGCTGGATCAGCTG    660

Clon             187 --------------------------------------------------    186

OmpA325minimd    661 TATAGCCAGCTGAGCAACCTGGATCCGAAAGATGGCAGCGTGGTGGTGCT    710

Clon             187 --------------------------------------------------    186

OmpA325minimd    711 GGGCTATACCGATCGCATTGGCAGCGATGCGTATAACCAGGGCCTGAGCG    760

Clon             187 --------------------------------------------------    186

OmpA325minimd    761 AACGCCGCGCGCAGAGCGTGGTGGATTATCTGATTAGCAAAGGCATTCCG    810

Clon             187 --------------------------------------------------    186

OmpA325minimd    811 GCGGATAAAATTAGCGCGCGCGGCATGGGCGAAAGCAACCCGGTGACCGG    860

Clon             187 --------------------------------------------------    186

OmpA325minimd    861 CAACACCTGCGATAACGTGAAACAGCGCGCGGCGCTGATTGATTGCCTGG    910

Clon             187 --------------------------------------------------    186

OmpA325minimd    911 CGCCGGATCGCCGCGTGGAAATTGAAGTGAAAGGCATTAAAGATGTGGTG    960

Clon             187 --------------------------------------------------    186

OmpA325minimd    961 ACCCAGCCGCAGGCG    975
```

```
Clon             187 ---------------    186
```

Clone 16

```
#=======================================

OmpA325minimd      1 GCGCCGAAAGATAACACCTGGTATACCGGCGCGAAACTGGGCTGGAGCCA     50
                                                                       
Clon               1 --------------------------------------------------      0

OmpA325minimd     51 GTATCATGATACCGGCTTTATTAACAACAACGGCCCGACCCATGAAAACC    100
                                                                       
Clon               1 --------------------------------------------------      0

OmpA325minimd    101 AGCTGGGCGCGGGCGCGTTTGGCGGCTATCAGGTGAACCCGTATGTGGGC    150
                                                                       
Clon               1 --------------------------------------------------      0

OmpA325minimd    151 TTTGAAATGGGCTATGATTGGCTGGGCCGCATGCCGTATAAAGGCAGCGT    200
                                                                       
Clon               1 --------------------------------------------------      0

OmpA325minimd    201 GGAAAACGGCGCGTATAAAGCGCAGGGCGTGCAGCTGACCGCGAAACTGG    250
                                                                       
Clon               1 --------------------------------------------------      0

OmpA325minimd    251 GCTATCCGATTACCGATGATCTGGATATTTATACCCGCCTGGGCGGCATG    300
                                                                       
Clon               1 --------------------------------------------------      0

OmpA325minimd    301 GTGTGGCGCGCGGATACCAAAAGCAACGTGTATGGCAAAAACCATGATAC    350
                                                                  ||.||
Clon               1 ---------------------------------------------GAAAC      5

OmpA325minimd    351 CGGCG-TGAGCCCGGTGTTTGCGGGCGGCGTGGAATATGCGATTACCCCG    399
                     | ||| |||.|                       ||    ||||||||||
Clon               6 C-GCGATGACC-----------------------AT----GATTACCCCG     27

OmpA325minimd    400 GAAATTGCGACCCGCCTGGAATATCAGTGGACCAACAACATTGGCGATGC    449
                                           |.|||.|.||         ||.|..|| 
Clon              28 ----------------------AGCAGCGAAC---------TGACCCTG-     45


OmpA325minimd    450 GCATACCATTGGCACCCGCCCGGATAACGGCATGCTGAGCCTGGGCGTGA    499
                         ||||..||||||.||||||    |||        |||||       
Clon              46 ----ACCAAAGGCACCAGCCCGG----CGG--------GCCTG-------     72

OmpA325minimd    500 GCTATCG-CTTTGGCCAGGGCGAAGCGGCGCCGGTGGTG-GCGCCGGCGC    547
                        |.|| .||||                  ||.||||| |||     ||
Clon              73 ---AACGAATTTG------------------CGCTGGTGAGCG-----GC     96

OmpA325minimd    548 CGGCGCCGGCGCCGGAAGTGCAGACCAAACATTT--TACCCTGAAAAGCG    595
                     |.|                            |||  ||||  |||     
Clon              97 CAG----------------------------TTTCATACC--GAA-----    111

OmpA325minimd    596 ATGTGCTGTTTAACTTTAACAAAGCGACCCTGAAACCGGAAGGCCAGGCG    645
                             ||||  ||                   |.||||.||||.|||
Clon             112 --------TTTA--TT-------------------CAGGAATGCCATGCG    132

OmpA325minimd    646 --GCGCTGGATCAGCTGTATAGCCAGCTGAGCAACCTGGATCCGAA---A    690
                       |.||.|.||..||.|                        |||.|   |
Clon             133 CAGGGCCGCATTCGCGG------------------------CCGCATTCA    158

OmpA325minimd    691 GATGGCAGCGTGGTGGTGCTGGGCTATACCG---ATCGCATTGGCAGCGA    737
                     |.|.|| || |||||||||||...|.|||||   ..|||.||        
Clon             159 GTTTGC-GC-TGGTGGTGCTGCAGTTTACCGGCCGCCGCTTT--------    198

OmpA325minimd    738 TGCGTATAACCAGGGCCTGAGCGAACGCCGCGCGCAGAGCGTGGTGGATT    787
```

```
Clon              199 --------------------------------------------------    198

OmpA325minimd     788 ATCTGATTAGCAAAGGCATTCCGGCGGATAAAATTAGCGCGCGCGGCATG    837

Clon              199 --------------------------------------------------    198

OmpA325minimd     838 GGCGAAAGCAACCCGGTGACCGGCAACACCTGCGATAACGTGAAACAGCG    887

Clon              199 --------------------------------------------------    198

OmpA325minimd     888 CGCGGCGCTGATTGATTGCCTGGCGCCGGATCGCCGCGTGGAAATTGAAG    937

Clon              199 --------------------------------------------------    198

OmpA325minimd     938 TGAAAGGCATTAAAGATGTGGTGACCCAGCCGCAGGCG    975

Clon              199 --------------------------------------    198
```

Clone 21

```
#=======================================

OmpA325minimd       1 GCGCCGAAAGATAACACCTGGTATACCGGCGCGAAACTGGGCTGGAGCCA     50

Clon                1 --------------------------------------------------      0

OmpA325minimd      51 GTATCATGATACCGGCTTTATTAACAACAACGGCCCGACCCATGAAAACC    100

Clon                1 --------------------------------------------------      0

OmpA325minimd     101 AGCTGGGCGCGGGCGCGTTTGGCGGCTATCAGGTGAACCCGTATGTGGGC    150

Clon                1 --------------------------------------------------      0

OmpA325minimd     151 TTTGAAATGGGCTATGATTGGCTGGGCCGCATGCCGTATAAAGGCAGCGT    200

Clon                1 --------------------------------------------------      0

OmpA325minimd     201 GGAAAACGGCGCGTATAAAGCGCAGGGCGTGCAGCTGACCGCGAAACTGG    250

Clon                1 --------------------------------------------------      0

OmpA325minimd     251 GCTATCCGATTACCGATGATCTGGATATTTATACCCGCCTGGGCGGCATG    300

Clon                1 --------------------------------------------------      0

OmpA325minimd     301 GTGTGGCGCGCGGATACCAAAAGCAACGTGTATGGCAAAAACCATGATAC    350

Clon                1 --------------------------------------------------      0

OmpA325minimd     351 CGGCGTGAGCCCGGTGTTTGCGGGCGGCGTGGAATATGCGATTACCCCGG    400

Clon                1 --------------------------------------------------      0

OmpA325minimd     401 AAATTGCGACCCGCCTGGAATATCAGTGGACCAACAACATTGGCGATGCG    450

Clon                1 --------------------------------------------------      0

OmpA325minimd     451 CATACCATTGGCACCCGCCCGGATAACGGCATGCTGAGCCTGGGCGTGAG    500

Clon                1 --------------------------------------------------      0

OmpA325minimd     501 CTATCGCTTTGGCCAGGGCGAAGCGGCG-CCGGTGGTGGCGCCGGCGCCG    549
                                          ||.||.|| |||||
Clon                1 --------------------AAACGCCGCCCGGT----------------     14

OmpA325minimd     550 GCGCCGGCGCCGGAAGTGC-AGACCAAACATTTTACCCTGAAAAGCGATG    598
                                  |||.||| |.||||..||||.|..|       |||
Clon               15 ------------GAACTGCAACACCACCCATTATCGC-------GCG---     42

OmpA325minimd     599 TGCTGTTTAACTTTAACAAAGCGACCCTGAAACCGGAAGGCCAGGCGGCG    648
```

```
                         |||.||       ||.||                     |||
Clon              43 --------AACATT-------CGCCC---------------------GCG     56

OmpA325minimd    649 CTGGATCAGCTGTATAGCCAGCTGAGCAACCTGGATCCGAAAGATGGCAG    698
                     |   ||        |.|||.| ||.|   |.|||        .||.||||
Clon              57 C---AT--------TCGCCCG-TGGG---CGTGG--------CATAGCAG     83

OmpA325minimd    699 CGTGGTGGTGCTGGGCTATACCGATCGCATTGGCAGCGATGCGTATAACC    748
                     ||||....|||||.||                        |||.||||
Clon              84 CGTGAAACTGCTGAGC------------------------GCGCATAA--    107

OmpA325minimd    749 AGGGCCTGAGCGAA---------CGCCGCGCGCAGAGCGTGGTGGATTAT    789
                     ||       |||||         .|||||.| |||..|||
Clon             108 AG-------GCGAATTTGTGACCTGCCGCAC-CAGCCCGT----------    139

OmpA325minimd    790 CTGATTAGCAAAGGCATTCCGGCGGATAAAATTAGCGCGCGGCATGGG    839
                         |||||.|||||.||                    |||.|||
Clon             140 ----TTAGCGAAGGCTTT--------------------GCGTGGC-----    160

OmpA325minimd    840 CGAAAGCAACCCGGTGACCGGCAACACCTGCGATAACGTGAAACAGCGCG    889
                          ||||||      ..|||.|.|.||||..|         |.||||
Clon             161 -----GCAACC------ATGGCCATAGCTGCTTT---------CTGCGC-    189

OmpA325minimd    890 CGGCGCTGATTGATTGCCTGGCGCCGGATCGCCGCGTGGAAATTGAAGTG    939

Clon             190 --------------------------------------------------    189

OmpA325minimd    940 AAAGGCATTAAAGATGTGGTGACCCAGCCGCAGGCG    975

Clon             190 ------------------------------------    189
```

Clone 23

```
#=======================================

OmpA325minimd      1 GCGCCGAAAGATAACACCTGGTATACCGGCGCGAAACTGGGCTGGAGCCA     50

Clon               1 --------------------------------------------------      0

OmpA325minimd     51 GTATCATGATACCGGCTTTATTAACAACAACGGCCCGACCCATGAAAACC    100

Clon               1 --------------------------------------------------      0

OmpA325minimd    101 AGCTGGGCGCGGGCGCGTTTGGCGGCTATCAGGTGAACCCGTATGTGGGC    150

Clon               1 --------------------------------------------------      0

OmpA325minimd    151 TTTGAAATGGGCTATGATTGGCTGGGCCGCATGCCGTATAAAGGCAGCGT    200

Clon               1 --------------------------------------------------      0

OmpA325minimd    201 GGAAAACGGCGCGTATAAAGCGCAGGGCGTGCAGCTGACCGCGAAACTGG    250

Clon               1 --------------------------------------------------      0

OmpA325minimd    251 GCTATCCGATTACCGATGATCTGGATATTTATACCCGCCTGGGCGGCATG    300

Clon               1 --------------------------------------------------      0

OmpA325minimd    301 GTGTGGCGCGCGGATACCAAAAGCAACGTGTATGGCAAAAACCATGATAC    350

Clon               1 --------------------------------------------------      0

OmpA325minimd    351 CGGCGTGAGCCCGGTGTTTGCGGGCGGCGTGGAATATGCGATTACCCCGG    400

Clon               1 --------------------------------------------------      0

OmpA325minimd    401 AAATTGCGACCCGCCTGGAATATCAGTGGACCAACAACATTGGCGATGCG    450

Clon               1 --------------------------------------------------      0

OmpA325minimd    451 CATACCATTGGCACCCGCCCGGATAACGGCATGCTGAGCCTGGGCGTGAG    500
```

```
Clon                 1 --------------------------------------------------      0

OmpA325minimd      501 CTATCGCTTTGGCCAGGGCGAAGCGGCGCCGGTGGTGGCGCCGGCGCCGG    550

Clon                 1 --------------------------------------------------      0

OmpA325minimd      551 CGCCGGCGCCGGAAGTGCAGACCAAACATTTTACCCTGAAAAGCGATGTG    600

Clon                 1 --------------------------------------------------      0

OmpA325minimd      601 CTGTTTAACTTTAACAAAGCGACCCTGAAACCGGAAGGCCAGGCGGCGCT    650

Clon                 1 --------------------------------------------------      0

OmpA325minimd      651 GGATCAGCTGTATAGCCAGCTGAGCAACCTGGATCCGAAAGATGGCAGCG    700
                                                            |||     ||||
Clon                 1 -------------------------------------AAA-----CAGC-      7

OmpA325minimd      701 TGGTGGTGCTGGGCTATACCGATCGCATTGGCAGCGATGCGTATAACCAG    750
                          ||..||||.||                 |||    |||.|.||||.|
Clon                 8 ---TGCCGCTGCGC-----------------CAG----GCGCAGAACCCG     33

OmpA325minimd      751 GGCCTGAGCGAACGCCGCGCGCAGAGCGTGGTGGATTATCT---GATTAG    797
                       .||||||   ||.|||            |||||      ||   |.|.|.
Clon                34 AGCCTGA---AAGGCC------------TGGTG------CTGCAGGTGAC     62

OmpA325minimd      798 CAAAGGCATTCCGGCGGATAAAA---TTAGCGCGCGCGGCATGGGCGAAA    844
                       |||..||...|.|||||||||.|   |||.|        ||     |||
Clon                63 CAACAGCCCGCTGGCGGATAACAACTTTACC--------CA-----GAA-     98

OmpA325minimd      845 GCAACCCGGTGACCG-GCAACACCTGCGATAACGTGAAACAGCGCGCGGC    893
                        ||.||..||| ||| |     ||.|||    ||.| |||||| ||| .|
Clon                99 -CAGCCGCGTG-CCGTG-----CCCGCG----CGCG-AACAGC-CGC-CC    134

OmpA325minimd      894 GCTGATTGATTGCCTGGCGCCGGATCGCCGCGTGGAAATTGAAGTGAAAG    943
                       |||||   |..||.|         ||||| ||     |||   ||||...
Clon               135 GCTGA---ACAGCAT---------TCGCC-CG-----ATT---GTGAGCC    163

OmpA325minimd      944 GCATTAAAGATGTGGTGACCCA-------GCCGCAGGCG--    975
                       ||||||             |||       ||||  .||.
Clon               164 GCATTA-------------CCATTCATTGGCCG--AGCTTT    189
```
